# Author Correction: Microbial Metagenomes Across a Complete Phytoplankton Bloom Cycle: High-Resolution Sampling Every 4 Hours Over 22 Days

**DOI:** 10.1038/s41597-025-04445-7

**Published:** 2025-01-20

**Authors:** Brook L. Nunn, Emma Timmins-Schiffman, Miranda C. Mudge, Deanna L. Plubell, Gabriella Chebli, Julia Kubanek, Michael Riffle, William S. Noble, Elizabeth Harvey, Tasman A. Nunn, Tatiana Rynearson, Marcel Huntemann, Kurt LaButti, Brian Foster, Bryce Foster, Simon Roux, Krishnaveni Palaniappan, Supratim Mukherjee, T. B. K. Reddy, Chris Daum, Alex Copeland, I-Min A. Chen, Natalia N. Ivanova, Nikos C. Kyrpides, Tijana Glavina del Rio, Emiley A. Eloe-Fadrosh

**Affiliations:** 1 University of Washington, Department of Genome Sciences, Seattle, WA 98195 USA; 2 Georgia Institute of Technology, School of Biological Sciences and School of Chemistry & Biochemistry, Parker H. Petit Institute for Bioengineering and Bioscience, Atlanta, GA 30332 USA; 3 Department of Biological Sciences, University of New Hampshire, Durham, NH 03824 USA; 4 Garfield High School, Seattle Public Schools, Seattle, WA 98122 USA; 5 School of Oceanography, University of Rhode Island, Narragansett, Rhode Island USA; 6 DOE Joint Genome Institute, Lawrence Berkeley National Laboratory, Berkeley, CA USA

**Keywords:** Marine biology, Water microbiology, Marine biology, Water microbiology

Correction to: *Scientific Data* 10.1038/s41597-024-04013-5, published online 22 November 2024

In the version of this article initially published, two errors in authorship were made. First, Tatiana Rynearson of the School of Oceanography, University of Rhode Island was mistakenly omitted from the final author list. Second, Kurt LaButti of the Joint Genome Institute was mistakenly omitted from the final author list and replaces Alicia Clum due to a staffing change at the Joint Genome Institute. These authorship omissions were not identified until after the work had been published. We are updating the authorship to appropriately recognize the contributions of these authors. The error has been corrected in the PDF and HTML versions of the article.

