# Supplementary material for: Microbial Metagenomes Across a Complete Phytoplankton Bloom Cycle: High-Resolution Sampling Every 4 Hours Over 22 Days
Source: Sci Data. 2024 Nov 22;11:1270. doi: 10.1038/s41597-024-04013-5 (PMC11584781; doi:10.1038/s41597-024-04013-5)
Supplement: Supplementary file 1 — Supplementary Information [file 41597_2024_4013_MOESM1_ESM.docx]

**Supplementary Information**

Brook L. Nunn^1*^ ; Emma Timmins-Schiffman^1^ ; Miranda C Mudge^1^; Deanna Plubell^1^; Gabriella Chebli^2^; Julia Kubanek^2^; Michael Riffle^1^; William S. Noble^1^; Elizabeth Harvey^3^; Tasman A. Nunn^1,4^; Marcel Huntemann^5^; Alicia Clum^5^; Brian Foster^5^; Bryce Foster^5^; Simon Roux^5^; Krishnaveni Palaniappan^5^; Supratim Mukherjee^5^; T.B.K. Reddy^5^; Chris Daum^5^ ; Alex Copeland^5^ ; I-Min A. Chen^5^; Natalia N. Ivanova^5^; Nikos C. Kyrpides^5^; Tijana Glavina del Rio^5^ ; Emiley A. Eloe-Fadrosh^5^

^1^University of Washington, Department of Genome Sciences, Seattle, WA 98195

^2^Georgia Institute of Technology, School of Biological Sciences and School of Chemistry & Biochemistry, Parker H. Petit Institute for Bioengineering and Bioscience, Atlanta, GA 30332

^3^Department of Biological Sciences, University of New Hampshire, Durham, NH 03824

^4^Garfield High School, Seattle Public Schools, Seattle, WA 98122

^5^DOE Joint Genome Institute, Lawrence Berkeley National Laboratory, Berkeley, CA, USA

**Table of Contents Page Number**

Supplemental Figure 1……………………………………………………………………………….3-4

**Supplemental Figure 1. MODIS satellite imagery and on-site microscopy confirm the presence of two distinct phytoplankton blooms.** Representative satellite and microscopic images confirm pre-bloom and bloom start for two phytoplankton blooms, as well as mid-bloom and post-bloom of the first phytoplankton bloom. MODIS satellite images of near-surface chlorophyll a concentration (mg m-3) in waters surrounding East Sound, WA, USA. The MODIS Chlorophyll a product is available from both the Terra and Aqua satellites. The sensor and imagery resolution is 1 km, and the temporal resolution is daily. References: MODISA_L2_OC doi:10.5067/AQUA/MODIS/L2/OC/2018. Microscopy images of whole water collected on-site confirm presence of *Chaetoceros spp.* throughout field season.
